# Sox17 Promotes Cell Cycle Progression and Inhibits TGF-β/Smad3 Signaling to Initiate Progenitor Cell Behavior in the Respiratory Epithelium

**DOI:** 10.1371/journal.pone.0005711

**Published:** 2009-05-27

**Authors:** Alexander W. Lange, Angela R. Keiser, James M. Wells, Aaron M. Zorn, Jeffrey A. Whitsett

**Affiliations:** 1 Division of Pulmonary Biology, Cincinnati Children's Hospital Medical Center and the University of Cincinnati College of Medicine, Cincinnati, Ohio, United States of America; 2 Division of Developmental Biology, Cincinnati Children's Hospital Medical Center and the University of Cincinnati College of Medicine, Cincinnati, Ohio, United States of America; Helmholtz Zentrum München/Ludwig-Maximilians-University Munich, Germany

## Abstract

The Sry-related high mobility group box transcription factor Sox17 is required for diverse developmental processes including endoderm formation, vascular development, and fetal hematopoietic stem cell maintenance. Expression of Sox17 in mature respiratory epithelial cells causes proliferation and lineage respecification, suggesting that Sox17 can alter adult lung progenitor cell fate. In this paper, we identify mechanisms by which Sox17 influences lung epithelial progenitor cell behavior and reprograms cell fate in the mature respiratory epithelium. Conditional expression of Sox17 in epithelial cells of the adult mouse lung demonstrated that cell cluster formation and respecification of alveolar progenitor cells toward proximal airway lineages were rapidly reversible processes. Prolonged expression of Sox17 caused the ectopic formation of bronchiolar-like structures with diverse respiratory epithelial cell characteristics in alveolar regions of lung. During initiation of progenitor cell behavior, Sox17 induced proliferation and increased the expression of the progenitor cell marker Sca-1 and genes involved in cell cycle progression. Notably, Sox17 enhanced cyclin D1 expression *in vivo* and activated cyclin D1 promoter activity *in vitro*. Sox17 decreased the expression of transforming growth factor-beta (TGF-β)-responsive cell cycle inhibitors in the adult mouse lung, including *p15*, *p21*, and *p57*, and inhibited TGF-β1-mediated transcriptional responses *in vitro*. Further, Sox17 interacted with Smad3 and blocked Smad3 DNA binding and transcriptional activity. Together, these data show that a subset of mature respiratory epithelial cells retains remarkable phenotypic plasticity and that Sox17, a gene required for early endoderm formation, activates the cell cycle and reinitiates multipotent progenitor cell behavior in mature lung cells.

## Introduction

Proteins in the Sry-related high mobility group box (Sox) family of transcription factors regulate developmental processes and cell type specification in various organ systems. Sox proteins share homology in the HMG domain, which binds the consensus DNA sequence (A/T)(A/T)CAA(A/T)GG to regulate gene expression and mediates protein interactions with transcriptional cofactors [1]. During vertebrate embryogenesis, Sox17 is required for formation of the endoderm, which gives rise to the liver, pancreas, and epithelium of the gastrointestinal and respiratory tracts [2]–[5]. In addition, constitutive expression of Sox17 in human embryonic stem cells is sufficient to promote differentiation of definitive endoderm progenitors [6]. Studies on mice with targeted deletion of Sox17 have identified additional roles for Sox17 in the maintenance of fetal hematopoietic stem cells, cardiovascular development, and angiogenesis [7]–[9]. Sox17 expression is dynamically regulated during endoderm formation and the programming of embryonic stem cells toward respiratory epithelial cell lineages *in vitro* [10]–[12]. Although Sox17 is highly expressed in the endoderm and is required for its formation prior to the emergence of the lung primordium, it is not readily detected in the respiratory epithelium thereafter. While conditional misexpression of Sox17 in respiratory epithelial cells of the embryonic mouse lung disrupted branching and differentiation of proximal/distal epithelial cell types, expression of Sox17 in the adult lung epithelium induced the formation of hyperplastic cell clusters in the alveolar region that contained cells expressing markers characteristic of diverse proximal airway epithelial lineages [13]. Together, these findings are consistent with a role for Sox17 in regulating progenitor cell behavior and lineage specification in various tissues.

The ability to reprogram mature, differentiated cell types into alternative lineages has been the subject of significant recent interest. Although the mechanisms underlying cell lineage reprogramming remain poorly understood, this phenomenon is generally associated with ectopic expression or reactivation of genes important for embryonic development and organogenesis [14]. While epithelial cells in the mature lung are normally quiescent, subpopulations of endogenous cells within the respiratory epithelium possess the capacity to reenter the cell cycle, proliferate, and redifferentiate into multiple epithelial cell types with appropriate function and location along the proximal/distal lung axis following injury. This is consistent with the notion that subsets of mature respiratory epithelial cells maintain remarkable phenotypic plasticity despite their differentiation status. In support of this concept, several lung stem/progenitor cell candidates have been identified in distinct niches along the airway epithelium, including basal cells, toxicant-resistant “variant” Clara cells, and ciliated cells in the conducting airway, bronchoalveolar stem cells (BASCs) located at the bronchoalveolar duct junction (BADJ), and type II cells in the peripheral lung [15]–[17]. During embryogenesis, respiratory epithelial cells are derived from progenitors in the foregut endoderm and are specified prior to the morphological appearance of the lung buds [18]. Several families of transcription factors influence lung morphogenesis and differentiation of the diverse respiratory epithelial cell types, including TTF-1, GATA6, β-catenin/TCF/LEF, Forkhead (Fox), and Sox family members [19]. However, whether transcription factors important for lung development can also influence progenitor cell behavior in mature respiratory epithelial cells is not known.

The TGF-β pathway regulates diverse biological processes in multiple cell types, including cell proliferation, differentiation, apoptosis, and migration. Following activation of heteromeric type II and type I kinase receptor complexes by TGF-β ligands, intracellular signaling is initiated by phosphorylation of receptor-activated Smad proteins. Phosphorylated Smad2 and Smad3, the effectors of the TGF-β pathway, interact with Smad4 and translocate to the nucleus to regulate transcription of downstream target genes [20]. The cellular responses to TGF-β signaling are further influenced by the interaction of Smad proteins with cofactors to modulate transcriptional activity. TGF-β signaling inhibits proliferation in multiple epithelial cell types by influencing the expression of cell cycle regulatory proteins to induce arrest prior to the restriction point in G1. For example, Smad2, 3, and 4 directly activate the promoters of the cyclin-dependent kinase inhibitors *p15* and *p21*, which in turn inhibit cdk2/4/6-cyclin complex activity [21]–[23]. In addition to negative regulation of branching morphogenesis in the embryonic lung, TGF-β/Smad signaling and expression of cyclin-dependent kinase inhibitors blocks proliferation of mature alveolar type II cells in culture [24]–[28]. Further, alveolar epithelial cells from *p21^−/−^* mice have an increased proliferation rate and lung tumorigenesis is enhanced in adult mice heterozygous for a null mutation in *TGF-β1*, supporting a role for this pathway in maintaining quiescence in the normal respiratory epithelium *in vivo* [29]–[31]. Whether TGF-β/Smad signaling and cyclin-dependent kinase inhibitors contribute to negative regulation of progenitor cell activation and cell cycle reentry in the mature respiratory epithelium has not been determined.

The present study was undertaken to determine the mechanisms by which expression of Sox17 in mature respiratory epithelial cells influences progenitor cell behavior. Conditional expression of Sox17 in the adult mouse lung induced proliferation and reversibly reprogrammed alveolar cells to form structures with phenotypic and morphological characteristics of the proximal airway. During the induction of progenitor cell behavior, Sox17 increased expression of Sca-1, a progenitor cell marker, and stimulated cell proliferation in association with altered expression of cell cycle regulatory genes. In addition, Sox17 physically interacted with Smad3 and negatively regulated Smad3 DNA binding and TGF-β/Smad3 transcriptional responses. These findings provide insight into the ability to reactivate multipotent progenitor cell behavior and reprogram epithelial cell lineages in the lung.

## Results

### Sox17 reversibly induces progenitor cell behavior in the alveolar epithelium *in vivo*

**Figure 1:**
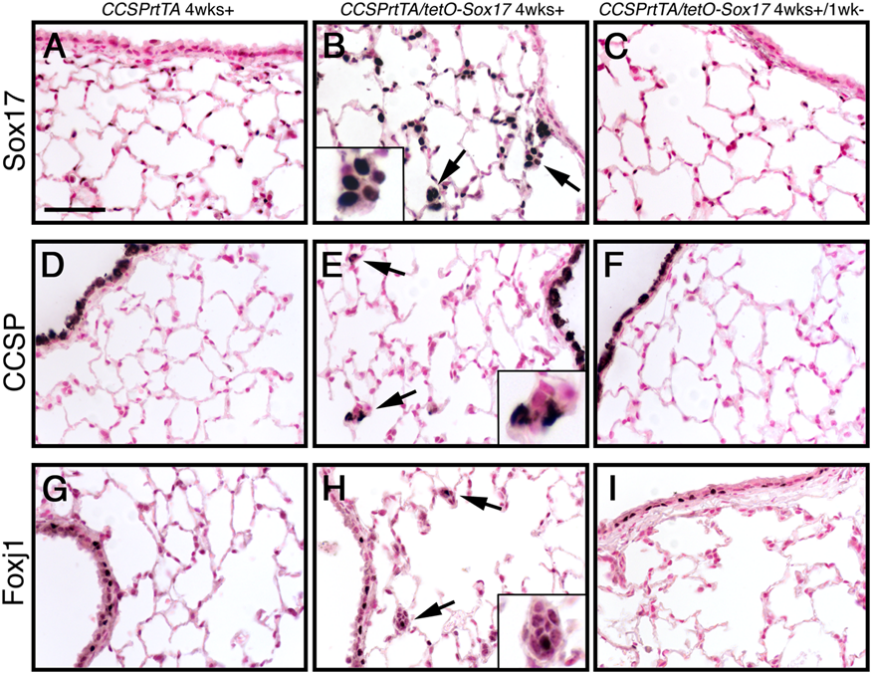
Sox17-induced cell clusters are reversible. Adult *CCSPrtTA* (A,D,G) and *CCSPrtTA/tetO-Sox17* (B,C,E,F,H,I) mice were maintained on Dox, and lungs were harvested after 4 weeks (wks) (A,B,D,E,G,H) or 1 week after discontinuing Dox (C,F,I). Immunostaining for Sox17 (A–C), CCSP (D–F), and Foxj1 (G–I) was performed on lung sections. (A) Sox17 staining was not observed in the airway epithelium in the absence of Dox. (B,E,H) Hyperplastic clusters of cells were observed in the alveolar region following Sox17 expression (arrows and insets). CCSP and Foxj1 staining was detected in a subset of the Sox17-induced alveolar cell clusters (arrows and insets; E,H). (C,F,I) Neither Sox17 transgene expression nor hyperplastic cell clusters were detected 1 week after removal from Dox. Scale bar, 50 µm.

Expression of Sox17 in peripheral respiratory epithelial cells of the adult mouse lung reprogrammed mature alveolar type II cells toward multiple proximal airway epithelial cell lineages, supporting a role in regulating plasticity/progenitor cell behavior [13]. To determine if alveolar cells become permanently respecified following ectopic Sox17 expression, Sox17 was conditionally expressed in respiratory epithelial cells of adult mice. Lungs from *CCSPrtTA/tetO-Sox17* and *CCSPrtTA* single transgenic control mice were harvested after 4 weeks exposure to doxycycline (Dox) or following removal of Dox for 1 week, and were examined for expression of Sox17 and the proximal airway epithelial cell markers CCSP (Clara cells) and Foxj1 (ciliated cells). Expression of Sox17 in peripheral respiratory epithelial cells induced the formation of atypical alveolar cell clusters which contained cells that expressed conducting airway epithelial cell markers CCSP and Foxj1 (Fig. 1B,E,H). In lungs from *CCSPrtTA/tetO-Sox17* mice in which Dox treatment was discontinued for 1 week prior to harvest, immunostaining for Sox17 was only detected in endothelial cells in the peripheral lung and was indistinguishable from controls (Fig. 1A,C). More notable, neither the alveolar cell clusters nor peripheral expression of CCSP or Foxj1 were observed in lungs from these mice (Fig. 1C,F,I). Together, these data demonstrate that the Sox17-induced alveolar cell cluster formation and lineage respecification are reversible processes and that continued expression of Sox17 is required to maintain progenitor cell behavior in adult *CCSPrtTA/tetO-Sox17* mice, revealing remarkable plasticity within a subset of mature respiratory epithelial cells.

### Bronchiolar-like lesions are induced in the alveoli following prolonged Sox17 expression

**Figure 2:**
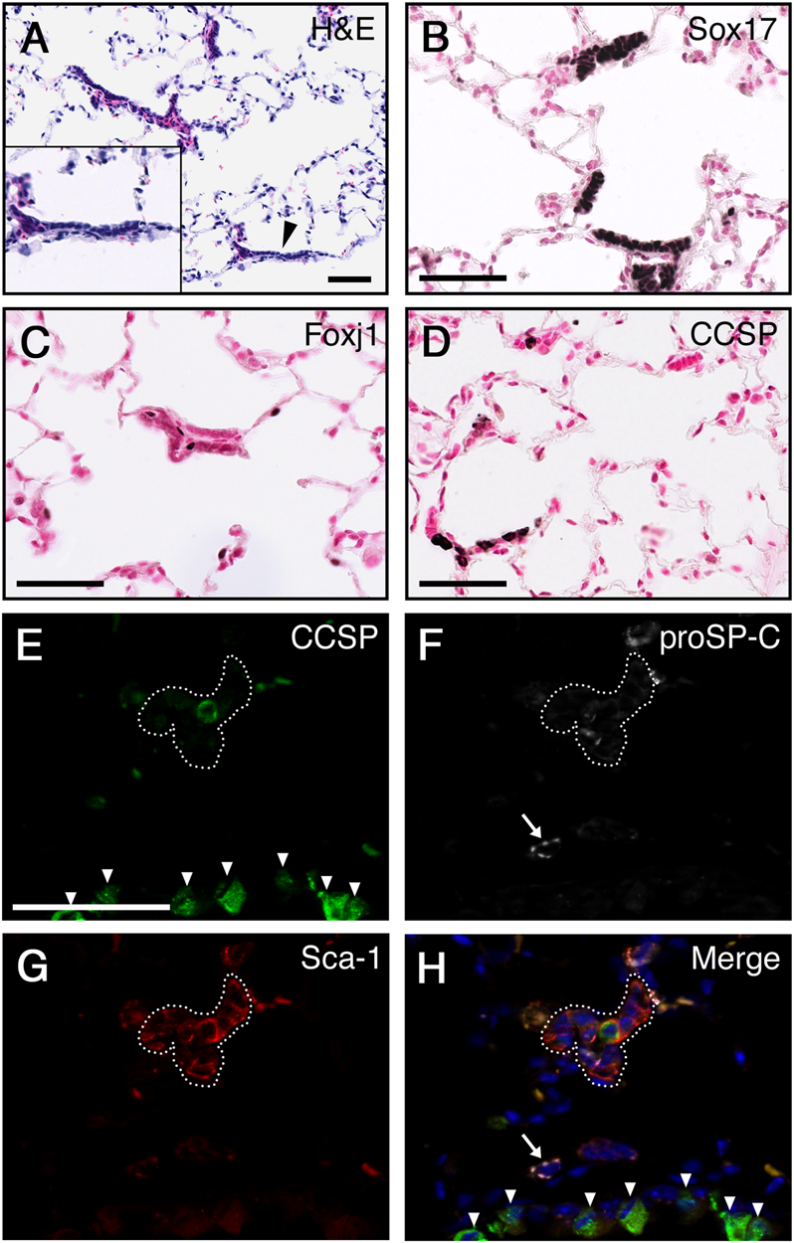
Prolonged expression of Sox17 results in formation of bronchiolar-like structures in the alveoli. Adult *CCSPrtTA/tetO-Sox17* transgenic mice were maintained on Dox for 12 months. (A) H&E staining shows the presence of bronchiolar-like sheets of cells in the peripheral lung. Arrowhead indicates the pleural surface. (B–D) Immunostaining for Sox17 (B), Foxj1 (C), and CCSP (D) was performed on lung sections. The Sox17-induced bronchiolar-like structures contained cells expressing proximal airway markers CCSP and Foxj1. (E–H) Immunofluorescent staining for CCSP (E), proSP-C (F), and Sca-1 (G). The bronchiolar-like structures (dotted outline) contained cells that express Sca-1 and subsets of cells expressing CCSP or proSP-C. Arrowheads demark CCSP-expressing cells in the bronchiolar epithelium and the arrow indicates normal proSP-C expression in a type II cell. Nuclei are stained with DAPI (H; blue) Scale bars, 50 µm.

To determine the effects of prolonged expression of Sox17 in respiratory epithelial cells, adult *CCSPrtTA/tetO-Sox17* mice were maintained on Dox for 12 months. Long-term expression of Sox17 caused the formation of organized sheets of epithelial cells in the peripheral lung with morphological similarities to the bronchiolar epithelium (Fig. 2). The bronchiolar-like structures expressed Sox17 (Fig. 2B) and contained subsets of cells that expressed proximal airway epithelial markers CCSP and Foxj1 (Fig. 2C–D), consistent with bronchiolar cell differentiation. While CCSP^+^ cells were detected in most of the bronchiolar-like structures, Foxj1^+^ cells were less frequently observed. Since the ability of Sox17 to reprogram mature alveolar type II cells suggests the induction of progenitor cell behavior, we examined the bronchiolar-like structures for coexpression of CCSP, proSP-C, and Sca-1, a property attributed to bronchoalveolar stem cells (BASCs), a potential lung stem/progenitor population [32]. Expression of Sca-1, a progenitor cell marker in several tissues, was detected in cells within the bronchiolar-like structures and colocalized with CCSP-expressing cells (Fig. 2E–H). While a rare subset of bronchiolar-like lesions contained cells that co-expressed CCSP and proSP-C (data not shown), CCSP^+^/proSP-C^+^/Sca-1^+^ cells were never observed. Thus, the Sox17-induced bronchiolar-like structures contained a mixed population of cells that expressed CCSP, Foxj1, Sca-1, CCSP^+^/Sca-1^+^, and CCSP^+^/proSP-C^+^, consistent with reprogramming of progenitor cells along several differentiated pathways. Such bronchiolar-like epithelial sheets were never detected in lungs from *CCSPrtTA* control mice maintained on Dox for 12 months (data not shown). Together these data show that prolonged expression of Sox17 in the adult mouse lung dramatically influences respiratory epithelial cell differentiation, generating ectopic structures in the peripheral lung with characteristics of the more proximal bronchiolar epithelium.

### Sox17 induces proliferation in adult respiratory epithelial cells

**Figure 3:**
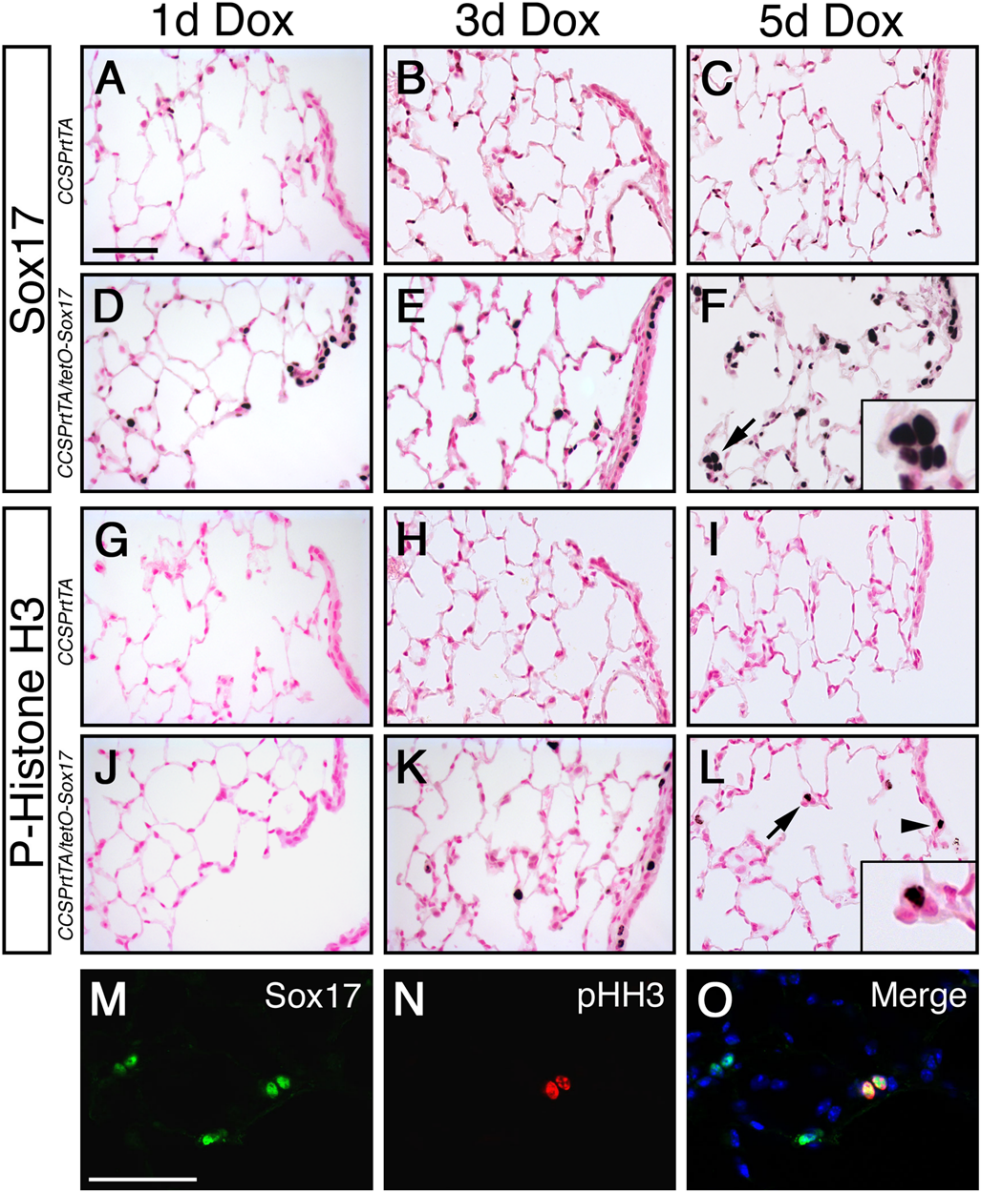
Sox17 induces proliferation of respiratory epithelial cells in the adult mouse lung. Immunohistochemistry was performed on lung sections from adult *CCSPrtTA* control (A–C; G–I) and *CCSPrtTA/tetO-Sox17* (D–F; J–L) transgenic mice after treatment with Dox for 1, 3, and 5 days (d). (A–F) Immunostaining shows endogenous expression of Sox17 in endothelial cells and Sox17 transgene expression in bronchioles and alveolar type II cells (D–F). Hyperplastic cell clusters are evident by 5 d (arrow and inset, F). (G–L) Phospho-histone H3 immunostaining shows respiratory epithelial cell proliferation after 3 d of Dox treatment in Sox17 transgenic mice. Proliferative cells were detected in the peripheral lung (K–L; arrow and inset) and bronchioles (K–L; arrowhead). (M–O) Colocalization of Sox17 (M) and phospho-histone H3 (pHH3; N) is shown by dual-label immunofluorescence after 5 d Dox exposure. Nuclei are stained with DAPI (O; blue). Scale bars, 50 µm.

To assess the molecular mechanisms by which Sox17 initiates cell cluster formation in respiratory epithelial progenitor cells, Sox17 was conditionally expressed in adult *CCSPrtTA/tetO-Sox17* mice for 1, 3, or 5 days. Endogenous expression of Sox17 was detected in endothelial cells in the peripheral lung but not in airway epithelial cells. In contrast, Sox17 staining was readily detected in bronchioles and alveolar type II cells of *CCSPrtTA/tetO-Sox17* mice, consistent with sites of rtTA-directed gene expression in this mouse line (Fig. 3A–F) [33]. The formation of cell clusters in the alveolar regions was evident 5 days after the induction of Sox17 expression (Fig. 3F). Immunostaining for phospho-histone H3 revealed the presence of mitotic cells in the both the bronchioles and alveoli as early as 3 days following Sox17 expression (Fig. 3G–L), with a 4.7-fold increase in the number of proliferative cells compared to control lungs (Fig. S1). Phospho-histone H3 staining was also observed in a subset cells at the bronchoalveolar duct junctions in the lungs of *CCSPrtTA/tetO-Sox17* mice (Fig. 3L; arrowhead). Although Sox17 expression and cell proliferation were detected in the bronchioles and bronchoalveolar duct junctions of *CCSPrtTA/tetO-Sox17* mice, hyperplastic foci within these regions were not evident. Dual immunofluorescence revealed colocalized expression of phospho-histone H3 with Sox17, demonstrating that proliferation occurs within the Sox17-expressing population of cells in *CCSPrtTA/tetO-Sox17* mice (Fig. 3M–O). Of the respiratory epithelial cells expressing Sox17, 28% coexpressed phospho-histone H3 (Fig. S1) and only a subset of alveolar type II cells expressing Sox17 formed cell clusters. Thus, Sox17 expression induces a subset of mature respiratory epithelial cells in the adult mouse lung to reenter the cell cycle, resulting in the formation of atypical cell clusters in the alveolar region within 5 days.

### Sox17 increases a Sca-1-positive cell population

**Figure 4:**
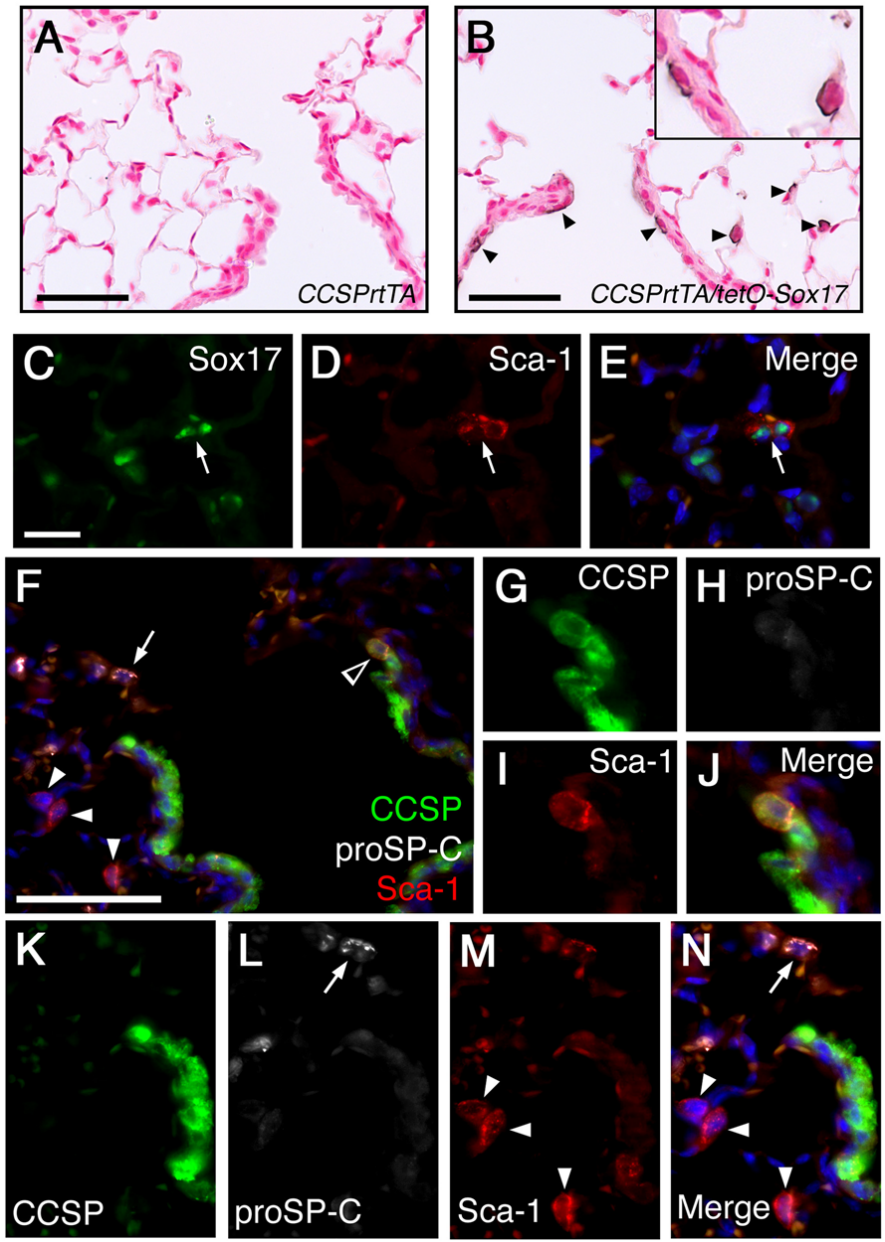
Sox17 increases Sca-1 expression adult respiratory epithelial cells. Adult *CCSPrtTA* control (A) and *CCSPrtTA/tetO-Sox17* (B–N) transgenic mice were exposed to Dox for 5 d. (A–B) Immunostaining for Sca-1 was performed on lung sections. Increased Sca-1 staining was detected in the peripheral lung and near the bronchoalveolar duct junctions after expression of Sox17 (arrowheads and inset). (C–E) Dual-label immunofluorescence for Sox17 (C) and Sca-1 (D) demonstrated that the Sca-1 positive cells coexpressed Sox17 (arrows). (F–N) Triple-label immunofluorescent staining was performed for CCSP, proSP-C, and Sca-1. Sca-1 colocalized with CCSP-expressing cells near the bronchoalveolar duct junctions (open arrowhead; magnified in G–J). Sca-1 positive cells located in the peripheral lung (arrowheads; magnified in K–N) did not coexpress proSP-C (arrow; F,L, and N). Scale bars, 50 µm (A–B; F), 20 µm (C–E).

Since Sox17 induced proliferation of alveolar type II cells and bronchiolar cells near the bronchoalveolar duct junctions, both considered lung stem/progenitor cell niches, expression of proposed pulmonary stem/progenitor cell markers was examined in *CCSPrtTA/tetO-Sox17* mice. Increased expression of Sca-1 was detected in the alveolar cells as well as in cells near the bronchoalveolar duct junction after 5 days of conditional Sox17 expression in *CCSPrtTA/tetO-Sox17* mice (Fig. 4A–B). Further, immunofluorescent double labeling demonstrated that the Sca-1 positive cells colocalized with Sox17-expressing cells in lungs of *CCSPrtTA/tetO-Sox17* mice (Fig. 4C–E). While the majority of the Sca-1-expressing cells did not show evidence of active proliferation, a rare subset of the Sca-1 positive cells coexpressed phospho-histone H3 (Fig. S2). Since Sox17 induced expression of Sca-1 in a subset of epithelial cells located in putative lung stem/progenitor cell niches in the alveolus and peripheral bronchioles, immunofluorescence was performed to determine if the population of Sca-1 positive cells coexpressed CCSP and/or proSP-C characteristic of BASCs. In lungs from *CCSPrtTA/tetO-Sox17* mice maintained on Dox for 5 days, colocalization of Sca-1 and CCSP was observed in cells along the bronchoalveolar duct junctions (Fig. 4F–N). In contrast, CCSP was not detected in the Sca-1-expressing cells detected in the alveolar region. In accordance with this observation, immunohistochemical analysis did not reveal any CCSP-expressing cells in the peripheral lung of *CCSPrtTA/tetO-Sox17* mice exposed to Dox for 5 days (data not shown). The Sox17-induced Sca-1 positive cells located in the alveolar region did not coexpress proSP-C (Fig. 4F–N). Further, CCSP^+^/proSP-C^+^/Sca-1^+^ cells were never detected at the bronchoalveolar duct junctions or in the lung periphery (n = 4 mice examining 3 sections from each lobe). Taken together, these studies show that the Sox17 induced Sca-1 expression in cells near the BADJ that coexpressed CCSP but not proSP-C, while the Sca-1 expressing cells induced in the alveolar region expressed neither CCSP nor proSP-C. Thus, while induction of Sca-1 supports the concept of progenitor cell activation, the molecular profile of the Sca-1-expressing population is distinct from that previously describe for BASCs [32], emphasizing the need for a better understanding of the phenotypic and functional characteristics of potential stem/progenitor cells in the lung.

### Sox17 influences expression of cell cycle regulatory genes

**Figure 5:**
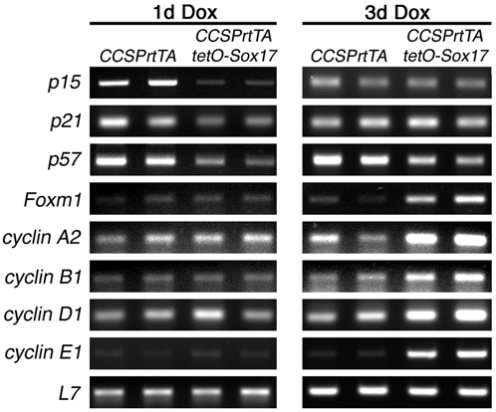
Sox17 regulates genes that control the cell cycle. RT-PCR was used to assess expression of cell cycle-related genes in lung tissue from adult *CCSPrtTA* and *CCSPrtTA/tetO-Sox17* mice treated with Dox for 1 or 3 days. Transcripts for the cyclin-dependent kinase inhibitors *p15*, *p21*, and *p57* were decreased in Sox17 transgenic lungs after 1 day of Dox and mRNAs for genes associated with cell cycle progression were increased by Sox17 after 3 days Dox exposure. *L7* was used as a loading control.

Since expression of Sox17 in respiratory epithelial cells in the adult mouse lung induced cell proliferation, the effects on cell cycle-associated gene expression was examined to identify potential downstream targets of Sox17 that regulate this process. Total RNA isolated from whole left lobes of adult *CCSPrtTA* control and *CCSPrtTA/tetO-Sox17* mice maintained on Dox for 2 and 3 days was used to analyze changes in cell cycle-related gene expression with a commercially available oligo SuperArray (data not shown). Subsequently, RT-PCR was used to confirm the changes in expression of a subset of the genes identified in the array. In addition to increased expression of *Sox17* (data not shown), mRNAs for the cyclin-dependent kinase inhibitors *p15*, *p21*, and *p57* were significantly decreased in lungs of *CCSPrtTA/tetO-Sox17* mice relative to *CCSPrtTA* controls following 1 day of exposure to Dox (Fig. 5). Whereas expression of *p57* was also decreased on lungs from *CCSPrtTA/tetO-Sox17* mice maintained on Dox for 3 days, expression of *p15* and *p21* was similar to controls. Further, while expression of *p19* was variably decreased in lungs from *CCSPrtTA/tetO-Sox17* mice maintained on Dox for 2 days, no differences in the expression of *p16* or *p27* were observed after expression of Sox17 for 1–3 days (data not shown). Consistent with the induction of proliferation observed by immunohistochemistry (Fig. 3), transcripts for genes that promote cell cycle progression, including *Foxm1*, *cyclin A2*, *cyclin B1*, *cyclin D1*, and *cyclin E1*, were increased in lungs of *CCSPrtTA/tetO-Sox17* mice after 3 days of Dox treatment (Fig. 5). Together, these results demonstrate that Sox17 expression in adult mouse lung results in decreased levels of cyclin-dependent kinase inhibitors associated with G1 arrest and increased expression of cell cycle-promoting genes, providing insight into the molecular mechanisms that regulate Sox17-induced proliferation associated with the initiation of progenitor cell behavior in respiratory epithelial cells.

### Sox17 induces cyclin D1 expression

**Figure 6:**
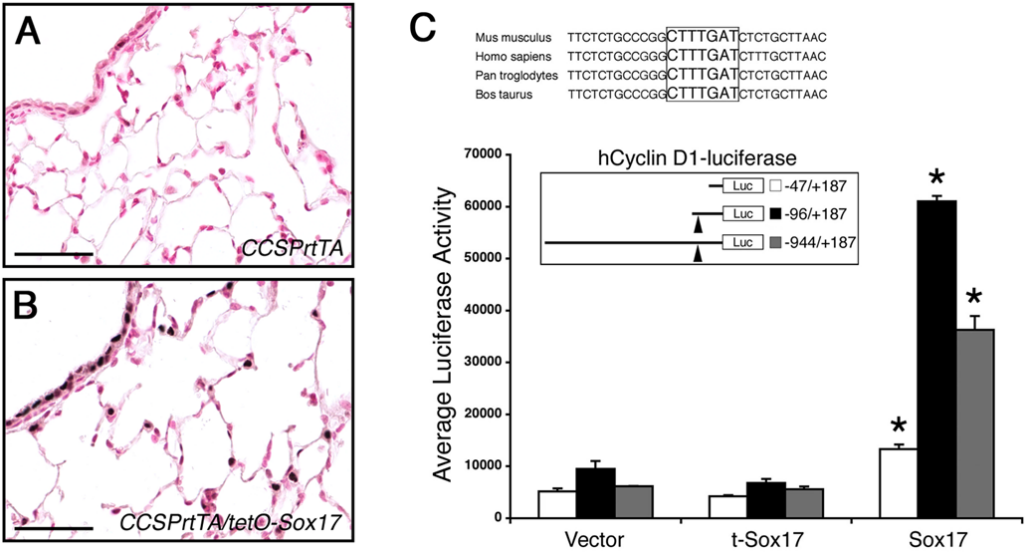
Cyclin D1 expression is induced by Sox17. (A–B) Immunohistochemistry for cyclin D1 was performed on lung sections from adult *CCSPrtTA* (A) and *CCSPrtTA/tetO-Sox17* (B) transgenic mice treated with Dox for 2 d. Cyclin D1 staining was increased in bronchioles and type II cells after Sox17 expression. Scale bars, 50 µm. (C) The regulatory region of *cyclin D1* contains a conserved Sox binding site (boxed). MLE-15 cells were transiently transfected with human *cyclin D1-luciferase* reporter constructs and empty vector, t-Sox17, or full length Sox17. While the −47/+187 *cyclin D1-luciferase* reporter was moderately responsive to Sox17 (2.6-fold), Sox17 strongly activated the *cyclin D1* promoter constructs containing the conserved Sox binding site (arrowheads; −96/+187; 6.4-fold and −944/+187; 5.9-fold). Experiments were performed three times in triplicate and representative results are shown±the standard deviation of the mean. Asterisks indicate statistical significance determined by Student's t-test (p<0.05).

Since cyclin D1 is a key regulator of progression through the G1 phase of the cell cycle and was increased in lungs from *CCSPrtTA/tetO-Sox17* mice, we sought to determine if it was a direct downstream target of Sox17 during induction of respiratory epithelial cell proliferation. Quantitative real time RT-PCR performed using total RNA isolated from whole left lobes demonstrated that *cyclin D1* mRNA was increased 1.3-fold in lungs from adult *CCSPrtTA/tetO-Sox17* mice exposed to Dox for 1 day (data not shown). Staining for cyclin D1 was markedly increased in bronchioles and alveolar type II cells of *CCSPrtTA/tetO-Sox17* mice following 2 days exposure to Dox (Fig. 6A–B). Thus, cyclin D1 mRNA and protein levels were significantly increased in lungs from *CCSPrtTA/tetO-Sox17* mice just prior to the onset of proliferation, consistent with the concept that cyclin D1 contributes to the cell cycle reentry of mature respiratory epithelial cells following expression of Sox17.

To determine if *cyclin D1* is a direct transcriptional target Sox17, the regulatory region of *cyclin D1* was examined for putative Sox binding sites. A well-conserved consensus Sox binding sequence was identified in *cyclin D1* proximal promoter, located at approximately −74 bp relative to exon 1 in the human and mouse genomic sequences (Fig. 6C). The functional significance of this potential Sox site was assessed by reporter assay using a human *cyclin D1* promoter deletion series. While the −47/+187 *cyclin D1-luciferase* reporter was moderately responsive to Sox17, the *cyclin D1-luciferase* constructs that contained the consensus Sox binding sequence were markedly activated by Sox17 (Fig. 6C). A truncated Sox17 isoform (t-Sox17), which lacks most of the HMG box and cannot bind DNA [34], did not affect *cyclin D1* promoter activity (Fig. 6C), indicating that activation of *cyclin D1-luciferase* reporters by Sox17 requires DNA binding. Together with the *in vivo* data, these results support the concept that *cyclin D1* is a direct downstream target of Sox17 in respiratory epithelial cells of *CCSPrtTA/tetO-Sox17* mice.

### Sox17 inhibits TGF-β1-induced transcriptional activity

**Figure 7:**
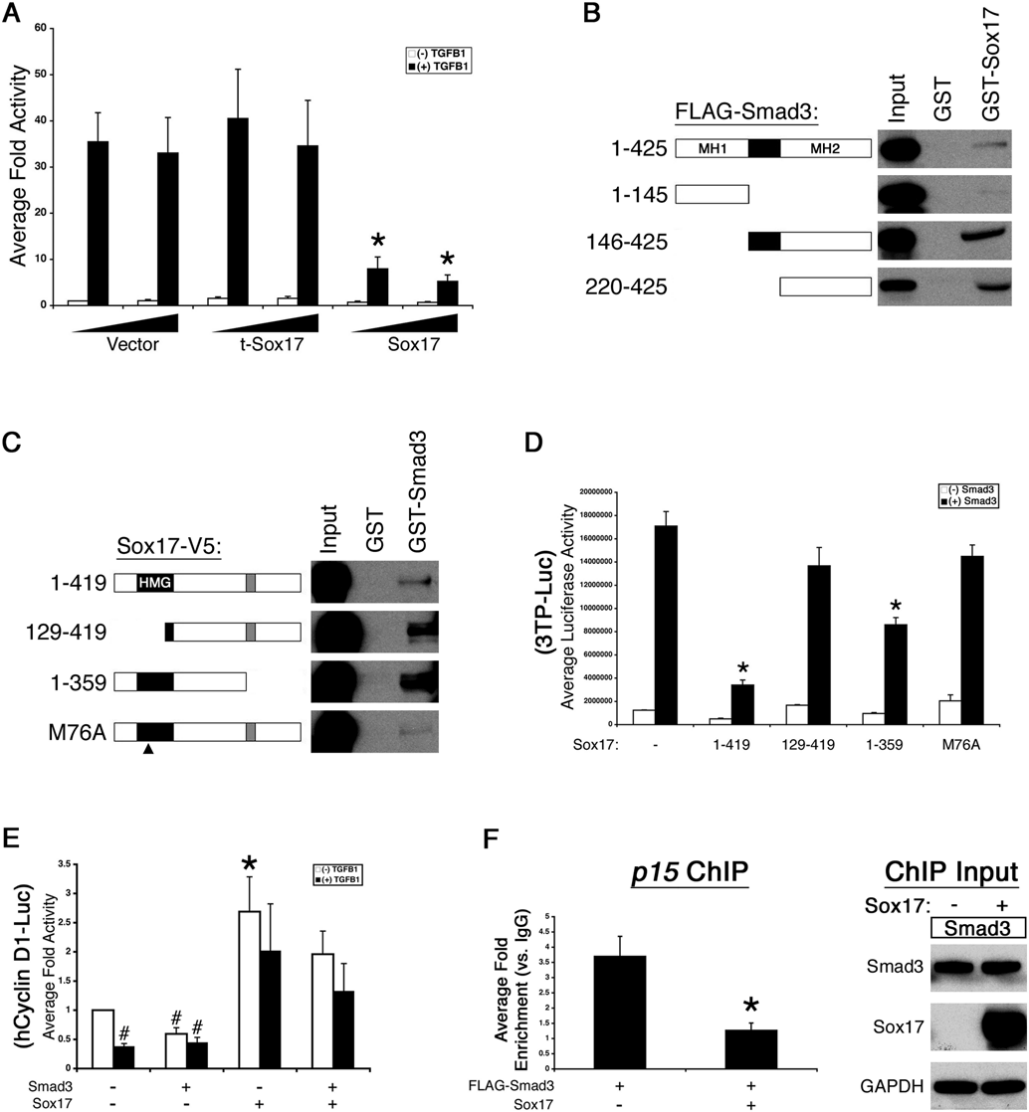
Sox17 interacts with Smad3 and inhibits TGF-β1/Smad3 transcriptional activity and Smad3 DNA binding. (A) MLE-15 cells were transfected with the TGF-β/Smad-responsive reporter *3TP-luciferase* (3TP-Luc) and increasing amounts of vector, t-Sox17, or full length Sox17 in the presence or absence 2 ng/ml TGF-β1. Sox17 inhibited TGF-β1-mediated activation of 3TP-Luc. The graph represents average fold activity±standard deviation. Asterisks indicate statistical significance determined by Student's t-test (p<0.05). (B–C) Lysates from MLE-15 cells expressing full length or mutant FLAG-Smad3 or Sox17-V5 (schematic representations) were incubated with GST only and GST-Sox17 (B) or GST-Smad3 (C), respectively. GST-pulldowns revealed an interaction between amino acids 129–359 of Sox17 and the linker region and MH2 domain of Smad3. MH1 and MH2, MAD homology domains; HMG, High mobility group. (D) MLE-15 cells were transfected with 3TP-Luc in the presence or absence Smad3 and wild type or mutant Sox17. Sox17 full length and C-terminal deletion inhibited Smad3-dependent transcriptional activity. Representative results are shown±standard deviation of the mean. Asterisks indicate statistical significance determined by Student's t-test (p<0.05). (E) Sox17 antagonizes TGF-β1/Smad3-mediated repression of *cyclin D1* promoter activity. MLE-15 cells were co-transfected with −944/+187 *cyclin D1-luciferase* and Smad3 or Sox17 in the presence or absence of 5 ng/ml TGF-β1. The graph represents average fold activity±standard deviation. Pound signs and asterisk indicate statistical significance determined by Student's t-test (p<0.05). (F) Sox17 blocks Smad3 DNA binding. MLE-15 cell were transfected with FLAG-Smad3 in the presence or absence of Sox17. After 24 h, cells were incubated with 5 ng/ml TGF-β1 for 8 h before harvesting. Binding of Smad3 to the *p15* promoter was assessed by chromatin immunoprecipitation and quantified by real time PCR. Graph represents average fold enrichment of FLAG immunoprecipitated samples relative to IgG negative control samples. Asterisk indicates statistical significance determined by Student's t-test (p<0.05). Expression of Smad3 and Sox17 inputs were assessed by immunoblot.

TGF-β is a potent inhibitor of proliferation in multiple epithelial cell types, including alveolar type II cells [28]. The mechanism underlying the anti-proliferative effects of TGF-β has been attributed, at least in part, to Smad-dependent transcriptional induction of cyclin-dependent kinase inhibitors including *p15* and *p21*, leading to G1 arrest [21]–[23]. Since expression of *p15* and *p21* was decreased in lungs of adult mice following Sox17 expression in respiratory epithelial cells, we sought to determine if Sox17 influences TGF-β-mediated transcriptional activity *in vitro* using the TGF-β/Smad responsive reporter *3TP-luciferase*. In MLE-15 cells, Sox17 markedly inhibited TGF-β1-induced *3TP-luciferase* reporter activity in a dose-dependent manner relative to vector control or t-Sox17 (Fig. 7A), demonstrating that Sox17 has an inhibitory effect on the TGF-β pathway.

### Sox17 interacts with Smad3 and inhibits Smad3-dependent transcriptional activity

Since Smad2/3 are the transcriptional effectors of TGF-β signaling, we examined if the inhibition of TGF-β1-induced transcriptional activity by Sox17 was mediated by an interaction with these Smads. GST-pulldown assays revealed a physical interaction between Sox17 and Smad3 (Fig. 7B–C). To identify which domains of Smad3 are required for the interaction, GST-pulldown assays were performed using deletion constructs for Smad3. While both the MAD homology domains (MH1 and MH2) of Smad3 interacted with Sox17 at low stringency binding conditions (data not shown), Smad3 1–145 did not bind to Sox17 under higher stringency conditions (Fig. 7B). Likewise, GST-pulldowns were performed using a series of Sox17 deletions and a point mutant, including a N-terminal deletion that removes 80% of the HMG domain (Sox17 129–419, which is equivalent to the t-Sox17 isoform), a C-terminal truncation that deletes the transactivation domain (Sox17 1–359), and a point mutation in the HMG domain (M76A), which is predicted to disrupt DNA binding but not protein structure [35]. Both of the Sox17 truncations and the point mutant maintained the ability to interact with Smad3 across multiple binding stringencies (Fig. 7C). Together, these data indicate that while multiple domains of Smad3 are capable of interacting with Sox17, the strongest binding is localized to the linker and MH2 regions from amino acids 146–425. Further, the data suggest that amino acids 129–359 of Sox17 mediate the interaction with Smad3. While the interaction observed between Sox17 and Smad3 was corroborated using co-immunoprecipitation assays, an interaction between Sox17 and Smad2 was not observed (data not shown).

To examine the functional significance of the interaction between Sox17 and Smad3 on Smad3 transcriptional activity, the *3TP-luciferase* reporter was co-transfected with Smad3 in the presence or absence of Sox17 in MLE-15 cells. Similar to its effect on TGF-β1 activity, Sox17 significantly inhibited Smad3-induced reporter activity (Fig. 7D). To identify the domains of Sox17 that mediate the repression of Smad3 transcriptional activity, reporter assays were performed using the Sox17 deletions and point mutant. While Sox17 1–359 maintained the ability to inhibit Smad3 activity, neither Sox17 129–419 nor Sox17 M76A inhibited Smad3 function (Fig. 7D). Immunocytochemistry demonstrated that full length Sox17 as well as the Sox17 deletions and point mutant all localized to the nucleus (Fig. S3). However, none of the Sox17 proteins influenced nuclear translocation of Smad3 in the presence or absence of TGF-β1 (Fig. S3), indicating that Sox17 does not antagonize Smad3 activity by regulating subcellular localization. Together, these data suggest that, although it is not required for binding to Smad3, the N-terminus of Sox17 is important for repression of Smad3 transcriptional activity.

To determine if the interaction between Sox17 and TGF-β/Smad3 signaling influences regulation of cell cycle-related genes, their effects on cell cycle gene promoters was examined. In MLE-15 cells, Sox17 alleviated repression of *cyclin D1* promoter activity by TGF-β1 and Smad3, consistent with an antagonistic effect of Sox17 on the TGF-β/Smad3 pathway (Fig. 7E). Since Sox17 negatively regulated Smad3 transcriptional activity in reporter assays but did not influence Smad3 nuclear import, we sought to determine if the repression was mediated by influencing Smad3 DNA binding. Smad3 regulates *p15* gene expression by directly binding to sites located within the first 113 bp of its promoter [21]. Using MLE-15 cells, Smad3 DNA binding to the *p15* promoter between −157 and +103 in the presence or absence of Sox17 was examined by chromatin immunoprecipitation and quantified by real time PCR. Sox17 significantly decreased Smad3 binding to the *p15* promoter (Fig. 7F). Together, these experiments show that Sox17 antagonizes Smad3 transcriptional activity by preventing its ability to bind DNA, providing a potential mechanism by which Sox17 decreased the expression of cell cycle inhibitors in the adult mouse lung. In addition, antagonizing TGF-β1/Smad3 repression of the *cyclin D1* promoter may also contribute to the Sox17-mediated induction of cyclin D1 expression observed *in vivo*.

## Discussion

Conditional expression of Sox17 in respiratory epithelial cells of the adult mouse lung induced proliferation and reversibly respecified alveolar type II epithelial cells to express markers characteristic of the differentiated bronchiolar epithelium, supporting the concept that a subset of mature respiratory epithelial cells possesses remarkable phenotypic plasticity and progenitor cell capabilities. Activation of this progenitor-like behavior in respiratory epithelial cells by Sox17 was associated with increased expression of Sca-1 and multiple genes that promote cell cycle reentry/progression. Sox17 decreased expression of cell cycle inhibitors *in vivo* and interacted with Smad3 to inhibit TGF-β1/Smad3-mediated transcriptional responses *in vitro*. Together, these data provide insight into the mechanisms by which Sox17 stimulates respiratory epithelial progenitor cell behavior and lineage respecification in the mature lung.

Expression of Sox17 reprogrammed a subset of mature alveolar type II cells to ectopically express markers characteristic of diverse conducting airway cell lineages, including ciliated, non-ciliated secretory cells, and goblet cells that are not normally detected in the alveolar regions of the lung [13], and led to the formation of highly organized bronchiolar-like structures in the peripheral lung. These results indicate that epithelial cells in the adult lung can serve as multipotent progenitors capable of lineage respecification. In support of this concept, conditional expression of SPDEF, an ETS family transcription factor, in respiratory epithelial cells of adult mice converts Clara cells into goblet cells [36]. The notion that Sox proteins have important functions in lineage reprogramming is further supported by the contribution of Sox2 toward induced pluripotency in somatic cells [37]–[40]. While the mechanisms that govern progenitor cell activation and cell fate respecification remain poorly understood, further analysis of Sox17-induced respiratory epithelial progenitors may provide insight into such processes in the mature lung.

Lineage reprogramming of adult cell types involves the expression of key developmental regulatory genes [14]. The finding from previous studies that expression of several transcription factors involved in regulating proximal/distal epithelial cell differentiation in the lung, including TTF-1, Foxa1, Foxa2, and β-catenin, is also increased in the Sox17-induced cell clusters suggests that Sox17 can reinitiate a program typical of the developing lung [13]. During endoderm formation in Xenopus, Sox17 regulates expression of *Foxa1* and *Foxa2*, and acts upstream of GATA6 [41]. Foxa1, Foxa2, and GATA6 influence the expression of differentiated respiratory epithelial cell markers, including CCSP and Foxj1 [42]–[44]. Taken together, these data support the concept that Sox17, which is necessary for endoderm formation, functions upstream of a hierarchy of transcription factors that cooperate in specification of endodermal cells from which respiratory epithelial lineages are derived. Constitutive expression of Sox17 in human embryonic stem cells is sufficient to commit cells to the definitive endoderm lineage, and increased expression of Sox17 is associated with the differentiation of embryonic stem cells toward cells with characteristics of the respiratory epithelium [5], [6], [10]–[12]. Together, these findings further support an early role for Sox17 in the establishment of endodermal cells that later serve as precursors of lung epithelial lineages.

The induction of proliferation in a subset of bronchiolar cells and alveolar type II cells by Sox17 was associated with increased expression of several cyclin genes known to stimulate cell cycle progression. Among the various cyclin genes induced by Sox17, only cyclin D1 functions in G1 prior to the restriction point of the cell cycle [45], [46]. Notably, forced expression of cyclin D1 along with cdk4 is sufficient to reinitiate cell cycle progression in multiple post-mitotic cell types [47], [48]. Therefore, the induction of cyclin D1 by Sox17 is likely to play an important role in stimulating mature respiratory epithelial cells to reenter the cell cycle. Our *in vitro* studies demonstrated that Sox17 directly activated the human *cyclin D1* promoter through a conserved site located at −74 bp relative to the first exon. Given the homology between their HMG domains, Sox and TCF/LEF proteins have similar preferences for consensus binding sequences, and previous studies have demonstrated that *cyclin D1* is directly regulated by β-catenin/TCF/LEF through this same site [49], [50]. In breast cancer cells, Sox2 interacts with β-catenin to cooperatively regulate the *cyclin D1* promoter through the −74 bp binding site as well [51]. In addition, Sox17 and Sox4 respectively inhibit and enhance proliferation of colon carcinoma cells through physical interactions with β-catenin and TCF/LEF to modulate protein stability and transcriptional activity, and Sox6 interacts with β-catenin and HDAC1 to repress *cyclin D1* promoter activity and proliferation in insulinoma cells [52], [53]. Taken together, these findings suggest that Sox proteins and β-catenin/TCF/LEF complexes may compete for common DNA binding sites and that their effects on transcription and cell behavior may depend on relative expression levels, protein interactions, and/or cell type. Although it is unclear whether Sox17 is a transcriptional activator of *cyclin D1 in vivo*, our data support a potential mechanism by which Sox17 directly induces *cyclin D1* to promote proliferation of a subset of mature respiratory epithelial cells.

Recent studies have shown that post-mitotic cells can reenter the cell cycle by blocking the expression of cyclin-dependent kinase inhibitors, supporting a role for this protein family in maintaining the balance between quiescence and proliferation [54]. In the present study, Sox17 decreased the expression of *p15*, *p21*, and *p57* in adult mouse lungs, and inhibited TGF-β1/Smad3 transcriptional activity *in vitro*. In addition, Sox17 physically interacted with Smad3 and abrogated Smad3 binding to the *p15* promoter. These findings support a model in which Sox17 antagonizes TGF-β/Smad-dependent expression of cell cycle inhibitors in mature respiratory epithelial cells, facilitating G1 progression and cell reentry. In addition, Sox17 counteracted repression of the *cyclin D1* promoter by TGF-β1 and Smad3. While we favor the notion that Sox17 directly activates the *cyclin D1* promoter, it is possible that inhibiting TGF-β/Smad3-mediated repression of *cyclin D1* also contributes to its increased expression in *CCSPrtTA/tetO-Sox17* mouse lungs. By decreasing expression of inhibitors and increasing expression of positive regulators of the G1 phase of the cell cycle, Sox17 establishes conditions that favor reactivation of proliferation in mature, normally quiescent respiratory epithelial cells.

In the present study, Sox17 interacted with Smad3 and repressed TGF-β1/Smad3 transcriptional activity. Although both the MH1 and MH2 domains of Smad3 bound Sox17, the strongest interaction was localized to the linker region and MH2 domains. The linker region of Smad3 contains a transactivation domain and the MH2 domain modulates transcription, whereas the MH1 domain of Smad3 regulates DNA binding and transcription. Thus, Sox17 interaction with these domains is consistent with the antagonistic effects of Sox17 on Smad3 DNA binding and transcriptional activity. While that amino acids 129–359 of Sox17 appear to mediate binding to Smad3, the N-terminus of Sox17 is necessary to antagonize Smad3 activity. Inhibition of β-catenin/TCF transcriptional activity is also dependent on the N-terminus of Sox17, wherein the HMG domain is required for interaction with TCF factors to influence their protein stability [52], [55]. Together, these findings indicate that the N-terminus of Sox17 containing the HMG domain is important for influencing the transcriptional responses of signaling pathways and that there may be multiple regions of Sox17 that contribute to complex protein interactions.

Sox17 initiated progenitor cell behavior in respiratory epithelial cells when ectopically expressed at high levels in the adult lung. While these studies provide insight into the effects of Sox17 on reprogramming mature respiratory epithelial cells, it is unclear whether they reflect a physiological role during development or repair. Since Sox17 is highly expressed in the endoderm, which gives rise to the respiratory epithelium, Sox17 likely plays an early role in specification of endodermal precursor cells prior to emergence of the lung primordium. In the lung, Sox17 is expressed in mesenchymal cells during branching morphogenesis and in pulmonary endothelial cells later in development and in the adult [13]. However, Sox17 mRNA was not detected in respiratory epithelial cells and immunohistochemical staining for endogenous Sox17 was not observed in the lung epithelium in the present study [13]. A number of Sox family members are expressed at high levels in various cell types in the developing and mature lung, including Sox2, 4, 7, 9, 11, 17, and 18 [56]–[60]. However, whether Sox proteins have distinct or redundant functions during lung formation and repair remains to be elucidated. Our data shows that the ability of Sox17 to induce progenitor cell behavior is mediated, at least in part, by activating cyclin D1 and decreasing TGF-β-responsive cell cycle inhibitor expression to promote proliferation. As the transcriptional pathways that regulate cell proliferation and differentiation during lung formation are reactivated during regeneration of pulmonary cell lineages following injury, determining how Sox proteins and other transcription factors integrate signals from multiple pathways is important toward understanding the regulatory mechanisms that control lung development and homeostasis.

## Materials and Methods

### Mice

*rCCSPrtTA* and *(tetO)_7_CMV-Sox17-IRES-NucGFP* transgenic mice used for conditional expression of Sox17 in respiratory epithelial cells have been previously described [13], [33], [61]. Adult *rCCSPrtTA/(tetO)_7_CMV-Sox17-IRES-NucGFP* double transgenic and *rCCSPrtTA* single transgenic control mice, referred to as *CCSPrtTA/tetO-Sox17* and *CCSPrtTA*, respectively, were maintained on doxycycline-containing food (625 mg/kg; Harlan Teklad, Madison, WI) as described for specific experiments. Animals were housed in pathogen-free conditions according to protocols approved by the Institutional Animal Care and Use Committee at Cincinnati Children's Hospital Research Foundation. Mice were sacrificed by anesthesia using a mixture of ketamine, aceprozamine, and xylazine and exsanguination by severing the inferior vena cava and descending aorta. All experiments were performed using at least 3 animals per group.

### Immunohistochemistry and Immunofluorescence

Lungs of adult mice were inflated with 4% paraformaldehyde/phosphate-buffered saline (PBS), fixed by immersion overnight at 4°C, and processed according to standard protocols for paraffin embedding. Immunohistochemistry was performed using primary antibodies for guinea pig-anti Sox17 (1∶10,000), rabbit anti-phospho-histone H3 (1∶1000; Santa Cruz), rabbit anti-CCSP (1∶5000), rabbit anti-Foxj1 (1∶14,000), rabbit anti-cyclin D1 (1∶400; Abcam), and rat anti-Sca-1 (1∶250; BD Pharmingen). Briefly, sections (5 µm) were deparaffinized, rehydrated through a graded ethanol series, and endogenous peroxidase activity was inactivated in 1.5% H_2_O_2_ in methanol. Microwave antigen retrieval was performed (except for Sca-1) using 10 mM citrate buffer, pH 6.0 and sections were blocked for 1–2 h in 4% normal goat or donkey serum in PBS-0.1% Triton X-100 (PBST) followed by primary antibody incubation overnight at 4°C. Sections were then washed and incubated with biotinylated secondary antibodies (1∶200; Vector Labs) followed by incubation in ABC reagent (Vectastain Elite ABC kit; Vector Labs). Antigen localization was detected with nickel-diaminobenzidine and enhanced with Tris-Cobalt. Sections were counterstained with 0.1% of Nuclear Fast Red and coverslipped using Permount (Fisher Scientific).

Immunofluorescence was performed as described above with the omission of peroxidase treatment. For immunofluorescent double and triple labeling, primary antibodies for guinea pig anti-Sox17 (1∶1000), rabbit anti-phospho-histone H3 (1∶500), rat anti-Sca-1 (1∶50), rabbit anti-proSP-C (1∶500), and guinea pig anti-CCSP (1∶10,000) were used with fluorophore-conjugated secondary antibodies (Alexa Fluor-488, Alexa Fluor-568, and Alexa Fluor-688; Molecular Probes). Sections were mounted with Vectashield anti-fade reagent containing DAPI (Vector Labs). Brightfield and fluorescent images were obtained using a Zeiss Axioplan2 microscope equipped with AxioVision Software.

### RNA analysis

Total RNA was extracted from whole left lobes of adult mice using TRIzol reagent (Invitrogen) and RNeasy Mini Kit (Qiagen) according to the manufacturer's recommendations. Mouse Oligo GEArrays (SuperArray Bioscience Corporation) were used per the manufacturer's protocol to examine expression of cell cycle-related genes in lungs from adult *CCSPrtTA/tetO-Sox17* (n = 3) and *CCSPrtTA* (n = 3) mice maintained on Dox for 2 and 3 days. Reverse transcription reactions were performed with 2 µg of RNA and oligo(dT) primers using the SuperScript First-Strand Synthesis kit (Invitrogen). RT-PCR was performed using the following primers: *p15* 5′-AGG CTT CCT GGA CAC GCT TG-3′ and 5′-AGA TGG GGC TGG GGA GAA AG-3′; *p21* 5′-CGA AAA CGG AGG CAG ACC AG-3′ and 5′-TCC TGA CCC ACA GCA GAA GAG G-3′; *p57* 5′-AAG CGA ACA GGC AGG CAA G-3′ and 5′-TAG AAG GCG GGC ACA GAC TC-3′; *Foxm1* 5′-CAC TTG GAT TGA GGA CCA CTT-3′ and 5′-GTC GTT TCT GCT GTG ATT CC-3′; *cyclin A2* 5′-ACC AAG AGA ATG TCA ACC CCG-3′ and 5′-GGT GAA GGC AGG CTG TTT ACT G-3′; *cyclin B1* 5′-AGG GTC GTG AAG TGA CTG GAA AC-3′ and 5′-TTG GGC ACA CAA CTG TTC TGC-3′; *cyclin D1* 5′-CAC AAC GCA CTT TCT TTC CA-3′ and 5′-GAC CAG CCT CTT CCT CCA C-3′; *cyclin E1* 5′-GCA GGC GAG GAT GAG AGC AG-3′ and 5′-ATA ACC ATG GCG AAC GGA ACC-3′; *L7* 5′-GAA GCT CAT CTA TGA GAA GGC-3′ and 5′-AAG ACG AAG GAG CTG CAG AAC-3′.

### Plasmids

The following expression constructs have been previously described: pCIG-Sox17 and pCIG-t-Sox17 (truncated Sox17) [13]; Sox17-V5 epitope-tagged constructs encoding for wild type Sox17 (1–419), a N-terminal deletion equivalent to the t-Sox17 isoform (129–419), C-terminal deletion prior to the transactivation domain (1–359), and a DNA binding domain point mutant (M76A); pGEX-mouse Sox17 encoding for glutathione *S*-transferase (GST)-Sox17 fusion protein [52]. The *3TP-luciferase* TGF-β/Smad-responsive reporter plasmid [62] was obtained from Dr. Jeff Molkentin (Cincinnati Children's Hospital Research Foundation), and human *cyclin D1-luciferase* promoter constructs, based on GenBank Accession number Z29078, have been previously described and were acquired from Dr. Karen Knudsen [63]. The pGEX-human Smad3 construct (Addgene plasmid 12630) encoding for GST-Smad3 was generated in the lab of Dr. Rik Derynck (UCSF) [64], and the FLAG-tagged Smad3 expression vectors were generated by Dr. Joan Massague (Memorial Sloan-Kettering Cancer Center) (FLAG-Smad3 (1–425), Addgene plasmid 14052; FLAG-Smad3 (1–145), Addgene plasmid 14965; FLAG-Smad3 (146–425), Addgene plasmid 14966; FLAG-Smad3 (220–425), Addgene plasmid 14967) [65], [66].

### Cell culture and Reporter Assays

MLE-15 cells, an SV40 immortalized mouse lung epithelial cell line, were maintained in HITES medium [67]. Cells were seeded in 6-well culture plates at 1×10^5^ cells per well and transfected using FuGENE6 (Roche). Transfections included a pCMV-β-galactosidase expression vector as an internal control for transfection efficiency. Recombinant human TGF-β1 (R&D Systems) was added directly to the culture medium where indicated. Cells were harvested 24 h post-transfection and luciferase activity was measured using a Luciferase Assay System kit (Promega) and normalized to β-galactosidase activity. Experiments were performed three times in triplicate and statistical significance was determined by paired Student's t-test.

### GST pulldowns

The GST-Sox17 and GST-Smad3 fusion proteins (and GST only) were expressed in BL21 cells induced with 1 mM isopropyl β-D-1-thiogalactopyranoside (IPTG). Cell pellets were resuspended in lysis buffer (25 mM Tris, pH 8.0; 0.5 mM EDTA, 0.2 M NaCl; 1 mM DTT; 15 µl/ml protease inhibitor cocktail (Sigma); 0.1 mM PMSF), sonicated, and GST and GST fusion proteins were purified using glutathione sepharose beads (Amersham). Sox17-V5 and FLAG-Smad3 constructs were expressed in MLE-15 cells. Whole cell protein lysates were harvested after 48 hours in cell lysis buffer (20 mM Tris, pH 8.0; 1 mM EDTA; 100 mM NaCl; 0.5% NP-40; 5 µl/ml protease inhibitor cocktail (Sigma); 0.1 mM PMSF), and precleared using glutathione sepharose beads. A 10% volume of the precleared lysates was retained for experimental inputs, and the remaining lysate was incubated with 4–8 µg of GST, GST-Sox17, or GST-Smad3 beads. Non-interacting proteins were removed by several washes (20 mM Tris, pH 8.0; 1 mM EDTA; 300–500 mM NaCl; 0.5% NP-40; 5 µl/ml protease inhibitor cocktail (Sigma); 0.1 mM PMSF) and samples were eluted by boiling in Laemmli sample buffer containing β-ME. Bound proteins were analyzed by SDS-PAGE and immunoblot using rabbit anti-FLAG (1∶6000; Sigma) and mouse anti-V5-HRP (1∶5000; Invitrogen) antibodies.

### Chromatin Immunoprecipitation

MLE-15 cells were seeded at 1×10^6^ in 10 cm plates and co-transfected with FLAG-Smad3 and pCIG or pCIG-Sox17 using FuGENE6 (Roche). After 24 h, cells were switched to serum-free media and treated with 5 ng/ml rhTGF-β1 (R&D Systems) for 8 h. Crosslinking was performed by treating cells with 1% formaldehyde for 10 min at room temperature and was terminated by addition of 0.125 M glycine. After rinsing with cold 1× PBS, cells from 3 plates per condition were pooled and incubated in hypotonic buffer (10 mM HEPES, pH 7.8; 10 mM KCl; 1.5 mM MgCl_2_; 0.5% NP-40; 5 µl/ml protease inhibitor cocktail) for 30 min at 4°C. Pellets were collected by centrifugation and incubated in lysis buffer (50 mM Tris, pH8.0; 10 mM EDTA; 1% SDS; 5 µl/ml protease inhibitor cocktail) for 30 min at 4°C. Lysates were sonicated (Diagenode bioruptor) to shear DNA and cell debris was removed by centrifugation. Lysates were precleared with Protein A/G Plus beads (Santa Cruz), sonicated salmon sperm DNA, and BSA (1 µg/µl) for 2 h at 4°C. A 1% aliquot of precleared chromatin was removed for experimental inputs. Equal volumes of the remaining lysate were incubated with EZview Red anti-FLAG M2 beads (Sigma) or Protein A/G Plus beads and normal mouse IgG (1.5 µg) overnight at 4°C. Beads were washed once in dialysis buffer (50 mM Tris, pH8.0; 2 mM EDTA) and 4 times with wash buffer (100 mM Tris, pH8.0; 500 mM LiCl; 1% NP-40; 1% deoxycholic acid) followed by elution in 200 µl (50 mM NaHCO_3_; 1% SDS). DNA-protein complexes were reverse crosslinked by incubation in 0.3 M NaCl and RNase A at 65°C overnight. DNA was isolated by phenol/chloroform extraction and ethanol precipitation and resuspended in 50 µl H_2_0. Genomic DNA obtained from chromatin immunoprecipitations was analyzed by real time PCR using Sybr Green (Roche) and an Opticon Monitor II system (MJ Research). Primers for the mouse *p15* gene promoter were as follows: 5′-CCA CCC CGC CTA TTT GTC-3′ and 5′-CCG TGA GAT TGC TAC AGC C-3′. Amount of DNA immunoprecipitated with FLAG and IgG beads was calculated based on threshold cycle [C(t)] using the ΔC(t) method and normalized to input samples. Results are expressed as fold enrichment of FLAG immunoprecipitated samples relative to IgG controls. Statistical significance was determined by paired Student's t-test. Expression of Smad3 and Sox17 inputs was assessed by immunoblot using cell lysates from transfections done in parallel to chromatin immunoprecipitations.

## Supporting Information

Figure S1 Sox17 increases proliferation of respiratory epithelial cells in the adult mouse lung. (A) Immunostaining for phospho-histone H3 (pHH3) was performed on lung sections from adult *CCSPrtTA* control (n = 3) and *CCSPrtTA/tetO-Sox17* (n = 3) mice maintained on Dox for 3 days. Total positive cells were quantified from 21 random fields for morphometric analysis. The average number of pHH3-positive cells per field was increased 4.74-fold in lungs from *CCSPrtTA/tetO-Sox17* mice relative to controls. Asterisk indicates statistical significance determined by Student's t-test (p<0.05). (B) Dual immunofluorescence for Sox17 and pHH3 was performed on lung sections from adult *CCSPrtTA/tetO-Sox17* mice maintained on Dox for 3 and 5 days and positive stained cells were quantified from 20 random fields. Phospho-histone H3 was coexpressed in 28% of the Sox17-expressing respiratory epithelial cells.
(0.18 MB TIF)

Figure S2 A rare subset of Sox17-induced Sca-1 positive cells coexpress phospho-histone H3. Dual-label immunofluorescence for phospho-histone H3 (pHH3; A) and Sca-1 (B) was performed on lung sections from adult *CCSPrtTA/tetO-Sox17* mice maintained on Dox for 5 days. A rare subset of pHH3-positive cells (arrow) colocalized with the Sca-1-expressing cells induced by Sox17 (arrow and arrowhead; B). Nuclei are stained with DAPI. Scale bar, 20 µm.
(0.36 MB TIF)

Figure S3 Sox17 colocalizes with Smad3 in the nucleus. MLE15 cells were transfected with FLAG-Smad3 and V5-tagged Sox17 full length or mutant contructs. After 24 h, cells were maintained in the absence (A) or presence (B) of TGF-β1 (2 ng/ml) for 2 h and dual-label immunofluorescence was performed for the V5 (green) and FLAG (red) epitopes. Expression of all of the Sox17 constructs colocalized with Smad3 in the nucleus. Nuclei are stained with DAPI (blue). Scale bar, 20 µm.
(2.27 MB TIF)

## References

[1] Wilson M, Koopman P (2002). Matching SOX: partner proteins and co-factors of the SOX family of transcriptional regulators.. Curr Opin Genet Dev.

[2] Alexander J, Stainier DY (1999). A molecular pathway leading to endoderm formation in zebrafish.. Curr Biol.

[3] Clements D, Woodland HR (2000). Changes in embryonic cell fate produced by expression of an endodermal transcription factor, Xsox17.. Mech Dev.

[4] Hudson C, Clements D, Friday RV, Stott D, Woodland HR (1997). Xsox17alpha and -beta mediate endoderm formation in Xenopus.. Cell.

[5] Kanai-Azuma M, Kanai Y, Gad JM, Tajima Y, Taya C (2002). Depletion of definitive gut endoderm in Sox17-null mutant mice.. Development.

[6] Seguin CA, Draper JS, Nagy A, Rossant J (2008). Establishment of endoderm progenitors by SOX transcription factor expression in human embryonic stem cells.. Cell Stem Cell.

[7] Kim I, Saunders TL, Morrison SJ (2007). Sox17 dependence distinguishes the transcriptional regulation of fetal from adult hematopoietic stem cells.. Cell.

[8] Matsui T, Kanai-Azuma M, Hara K, Matoba S, Hiramatsu R (2006). Redundant roles of Sox17 and Sox18 in postnatal angiogenesis in mice.. J Cell Sci.

[9] Sakamoto Y, Hara K, Kanai-Azuma M, Matsui T, Miura Y (2007). Redundant roles of Sox17 and Sox18 in early cardiovascular development of mouse embryos.. Biochem Biophys Res Commun.

[10] Kubo A, Shinozaki K, Shannon JM, Kouskoff V, Kennedy M (2004). Development of definitive endoderm from embryonic stem cells in culture.. Development.

[11] Rippon HJ, Polak JM, Qin M, Bishop AE (2006). Derivation of distal lung epithelial progenitors from murine embryonic stem cells using a novel three-step differentiation protocol.. Stem Cells.

[12] Winkler ME, Mauritz C, Groos S, Kispert A, Menke S (2008). Serum-free differentiation of murine embryonic stem cells into alveolar type II epithelial cells.. Cloning Stem Cells.

[13] Park KS, Wells JM, Zorn AM, Wert SE, Whitsett JA (2006). Sox17 influences the differentiation of respiratory epithelial cells.. Dev Biol.

[14] Zhou Q, Melton DA (2008). Extreme makeover: converting one cell into another.. Cell Stem Cell.

[15] Besnard V, Whitsett JA, Lanza RP, Langer RS, Vacanti J (2007). Progenitor cells in the respiratory system.. Principles of Tissue Engineering (3rd edition).

[16] Kim CF (2007). Paving the road for lung stem cell biology: bronchioalveolar stem cells and other putative distal lung stem cells.. Am J Physiol Lung Cell Mol Physiol.

[17] Otto WR (2002). Lung epithelial stem cells.. J Pathol.

[18] Perl AK, Wert SE, Nagy A, Lobe CG, Whitsett JA (2002). Early restriction of peripheral and proximal cell lineages during formation of the lung.. Proc Natl Acad Sci U S A.

[19] Maeda Y, Dave V, Whitsett JA (2007). Transcriptional control of lung morphogenesis.. Physiol Rev.

[20] Massague J (2000). How cells read TGF-beta signals.. Nat Rev Mol Cell Biol.

[21] Feng XH, Lin X, Derynck R (2000). Smad2, Smad3 and Smad4 cooperate with Sp1 to induce p15(Ink4B) transcription in response to TGF-beta.. Embo J.

[22] Moustakas A, Kardassis D (1998). Regulation of the human p21/WAF1/Cip1 promoter in hepatic cells by functional interactions between Sp1 and Smad family members.. Proc Natl Acad Sci U S A.

[23] Pardali K, Kurisaki A, Moren A, ten Dijke P, Kardassis D (2000). Role of Smad proteins and transcription factor Sp1 in p21(Waf1/Cip1) regulation by transforming growth factor-beta.. J Biol Chem.

[24] Buckley S, Driscoll B, Anderson KD, Warburton D (1997). Cell cycle in alveolar epithelial type II cells: integration of Matrigel and KGF.. Am J Physiol.

[25] Corroyer S, Nabeyrat E, Clement A (1997). Involvement of the cell cycle inhibitor CIP1/WAF1 in lung alveolar epithelial cell growth arrest induced by glucocorticoids.. Endocrinology.

[26] Nabeyrat E, Corroyer S, Epaud R, Besnard V, Cazals V (2000). Retinoic acid-induced proliferation of lung alveolar epithelial cells is linked to p21(CIP1) downregulation.. Am J Physiol Lung Cell Mol Physiol.

[27] Bhaskaran M, Kolliputi N, Wang Y, Gou D, Chintagari NR (2007). Trans-differentiation of alveolar epithelial type II cells to type I cells involves autocrine signaling by transforming growth factor beta 1 through the Smad pathway.. J Biol Chem.

[28] Zhang F, Nielsen LD, Lucas JJ, Mason RJ (2004). Transforming growth factor-beta antagonizes alveolar type II cell proliferation induced by keratinocyte growth factor.. Am J Respir Cell Mol Biol.

[29] Blundell R, Harrison DJ, O'Dea S (2004). p21(Waf1/Cip1) regulates proliferation and apoptosis in airway epithelial cells and alternative forms have altered binding activities.. Exp Lung Res.

[30] Kang Y, Mariano JM, Angdisen J, Moody TW, Diwan BA (2000). Enhanced tumorigenesis and reduced transforming growth factor-beta type II receptor in lung tumors from mice with reduced gene dosage of transforming growth factor-beta1.. Mol Carcinog.

[31] Tang B, Bottinger EP, Jakowlew SB, Bagnall KM, Mariano J (1998). Transforming growth factor-beta1 is a new form of tumor suppressor with true haploid insufficiency.. Nat Med.

[32] Kim CF, Jackson EL, Woolfenden AE, Lawrence S, Babar I (2005). Identification of bronchioalveolar stem cells in normal lung and lung cancer.. Cell.

[33] Perl AK, Zhang L, Whitsett JA (2009). Conditional expression of genes in the respiratory epithelium in transgenic mice: cautionary notes and toward building a better mouse trap.. Am J Respir Cell Mol Biol.

[34] Kanai Y, Kanai-Azuma M, Noce T, Saido TC, Shiroishi T (1996). Identification of two Sox17 messenger RNA isoforms, with and without the high mobility group box region, and their differential expression in mouse spermatogenesis.. J Cell Biol.

[35] Weiss MA (2001). Floppy SOX: mutual induced fit in hmg (high-mobility group) box-DNA recognition.. Mol Endocrinol.

[36] Park KS, Korfhagen TR, Bruno MD, Kitzmiller JA, Wan H (2007). SPDEF regulates goblet cell hyperplasia in the airway epithelium.. J Clin Invest.

[37] Park IH, Zhao R, West JA, Yabuuchi A, Huo H (2008). Reprogramming of human somatic cells to pluripotency with defined factors.. Nature.

[38] Takahashi K, Tanabe K, Ohnuki M, Narita M, Ichisaka T (2007). Induction of pluripotent stem cells from adult human fibroblasts by defined factors.. Cell.

[39] Takahashi K, Yamanaka S (2006). Induction of pluripotent stem cells from mouse embryonic and adult fibroblast cultures by defined factors.. Cell.

[40] Yu J, Vodyanik MA, Smuga-Otto K, Antosiewicz-Bourget J, Frane JL (2007). Induced pluripotent stem cell lines derived from human somatic cells.. Science.

[41] Sinner D, Rankin S, Lee M, Zorn AM (2004). Sox17 and beta-catenin cooperate to regulate the transcription of endodermal genes.. Development.

[42] Keijzer R, van Tuyl M, Meijers C, Post M, Tibboel D (2001). The transcription factor GATA6 is essential for branching morphogenesis and epithelial cell differentiation during fetal pulmonary development.. Development.

[43] Liu C, Morrisey EE, Whitsett JA (2002). GATA-6 is required for maturation of the lung in late gestation.. Am J Physiol Lung Cell Mol Physiol.

[44] Wan H, Dingle S, Xu Y, Besnard V, Kaestner KH (2005). Compensatory roles of Foxa1 and Foxa2 during lung morphogenesis.. J Biol Chem.

[45] Blagosklonny MV, Pardee AB (2002). The restriction point of the cell cycle.. Cell Cycle.

[46] Donjerkovic D, Scott DW (2000). Regulation of the G1 phase of the mammalian cell cycle.. Cell Res.

[47] Latella L, Sacco A, Pajalunga D, Tiainen M, Macera D (2001). Reconstitution of cyclin D1-associated kinase activity drives terminally differentiated cells into the cell cycle.. Mol Cell Biol.

[48] Tamamori-Adachi M, Ito H, Sumrejkanchanakij P, Adachi S, Hiroe M (2003). Critical role of cyclin D1 nuclear import in cardiomyocyte proliferation.. Circ Res.

[49] Shtutman M, Zhurinsky J, Simcha I, Albanese C, D'Amico M (1999). The cyclin D1 gene is a target of the beta-catenin/LEF-1 pathway.. Proc Natl Acad Sci U S A.

[50] Tetsu O, McCormick F (1999). Beta-catenin regulates expression of cyclin D1 in colon carcinoma cells.. Nature.

[51] Chen Y, Shi L, Zhang L, Li R, Liang J (2008). The molecular mechanism governing the oncogenic potential of SOX2 in breast cancer.. J Biol Chem.

[52] Sinner D, Kordich JJ, Spence JR, Opoka R, Rankin S (2007). Sox17 and Sox4 differentially regulate beta-catenin/T-cell factor activity and proliferation of colon carcinoma cells.. Mol Cell Biol.

[53] Iguchi H, Urashima Y, Inagaki Y, Ikeda Y, Okamura M (2007). SOX6 suppresses cyclin D1 promoter activity by interacting with beta-catenin and histone deacetylase 1, and its down-regulation induces pancreatic beta-cell proliferation.. J Biol Chem.

[54] Pajalunga D, Mazzola A, Salzano AM, Biferi MG, De Luca G (2007). Critical requirement for cell cycle inhibitors in sustaining nonproliferative states.. J Cell Biol.

[55] Zhang W, Glockner SC, Guo M, Machida EO, Wang DH (2008). Epigenetic inactivation of the canonical Wnt antagonist SRY-box containing gene 17 in colorectal cancer.. Cancer Res.

[56] Dunn TL, Mynett-Johnson L, Wright EM, Hosking BM, Koopman PA (1995). Sequence and expression of Sox-18 encoding a new HMG-box transcription factor.. Gene.

[57] Gontan C, de Munck A, Vermeij M, Grosveld F, Tibboel D (2008). Sox2 is important for two crucial processes in lung development: branching morphogenesis and epithelial cell differentiation.. Dev Biol.

[58] Perl AK, Kist R, Shan Z, Scherer G, Whitsett JA (2005). Normal lung development and function after Sox9 inactivation in the respiratory epithelium.. Genesis.

[59] Sock E, Rettig SD, Enderich J, Bosl MR, Tamm ER (2004). Gene targeting reveals a widespread role for the high-mobility-group transcription factor Sox11 in tissue remodeling.. Mol Cell Biol.

[60] Takash W, Canizares J, Bonneaud N, Poulat F, Mattei MG (2001). SOX7 transcription factor: sequence, chromosomal localisation, expression, transactivation and interference with Wnt signalling.. Nucleic Acids Res.

[61] Perl AK, Tichelaar JW, Whitsett JA (2002). Conditional gene expression in the respiratory epithelium of the mouse.. Transgenic Res.

[62] Wrana JL, Attisano L, Carcamo J, Zentella A, Doody J (1992). TGF beta signals through a heteromeric protein kinase receptor complex.. Cell.

[63] Herber B, Truss M, Beato M, Muller R (1994). Inducible regulatory elements in the human cyclin D1 promoter.. Oncogene.

[64] Zhang Y, Feng X, We R, Derynck R (1996). Receptor-associated Mad homologues synergize as effectors of the TGF-beta response.. Nature.

[65] Kretzschmar M, Doody J, Timokhina I, Massague J (1999). A mechanism of repression of TGFbeta/Smad signaling by oncogenic Ras.. Genes Dev.

[66] Chen CR, Kang Y, Siegel PM, Massague J (2002). E2F4/5 and p107 as Smad cofactors linking the TGFbeta receptor to c-myc repression.. Cell.

[67] Wikenheiser KA, Vorbroker DK, Rice WR, Clark JC, Bachurski CJ (1993). Production of immortalized distal respiratory epithelial cell lines from surfactant protein C/simian virus 40 large tumor antigen transgenic mice.. Proc Natl Acad Sci U S A.

