# Supplementary figures and images for: Sox17 Promotes Cell Cycle Progression and Inhibits TGF-β/Smad3 Signaling to Initiate Progenitor Cell Behavior in the Respiratory Epithelium
Source: PLoS One. 2009 May 27;4(5):e5711. doi: 10.1371/journal.pone.0005711 (PMC2682659; doi:10.1371/journal.pone.0005711)

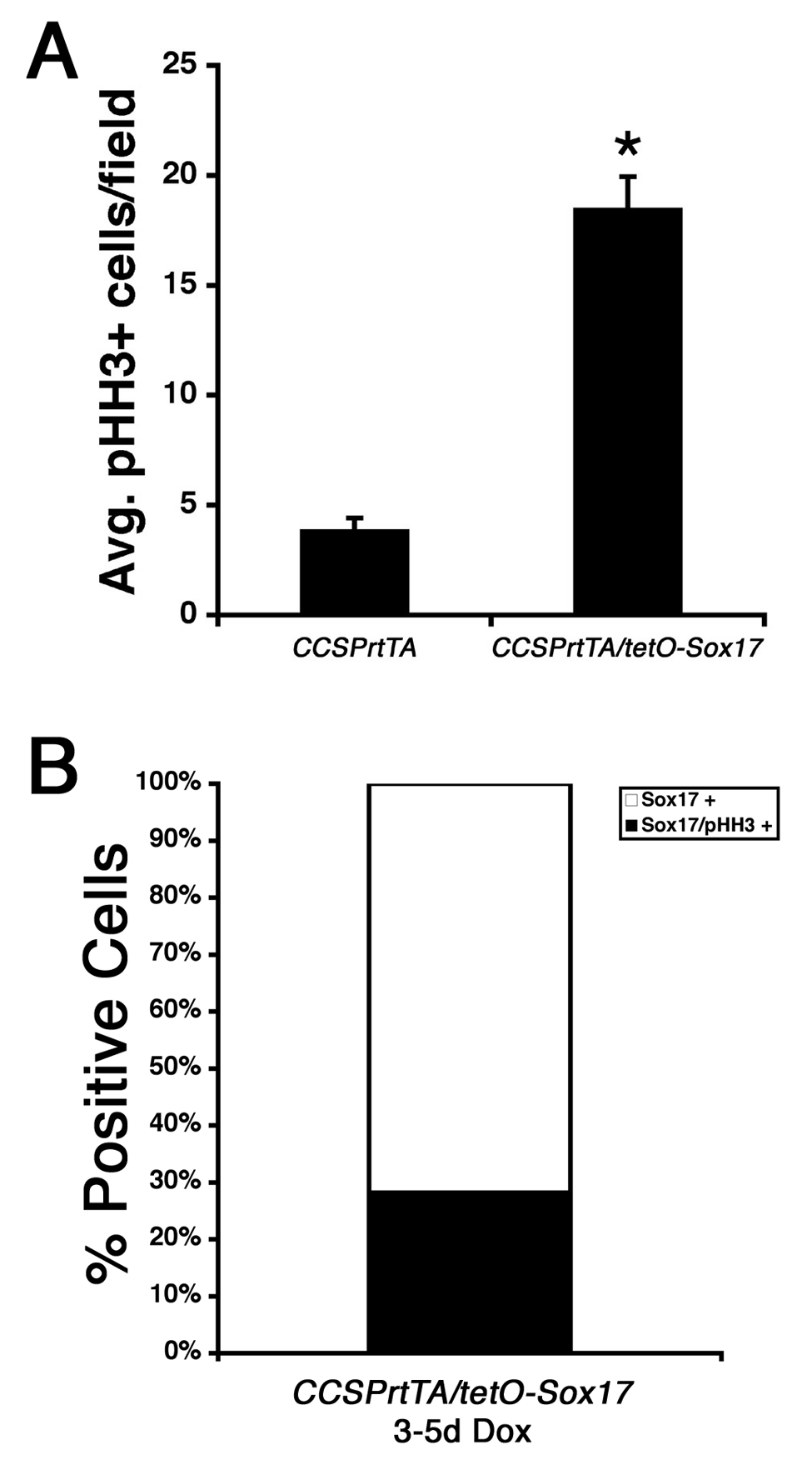

Supplement: Figure S1 — Sox17 increases proliferation of respiratory epithelial cells in the adult mouse lung. (A) Immunostaining for phospho-histone H3 (pHH3) was performed on lung sections from adult CCSPrtTA control (n = 3) and CCSPrtTA/tetO-Sox17 (n = 3) mice maintained on Dox for 3 days. Total positive cells were quantified from 21 random fields for morphometric analysis. The average number of pHH3-positive cells per field was increased 4.74-fold in lungs from CCSPrtTA/tetO-Sox17 mice relative to controls. Asterisk indicates statistical significance determined by Student's t-test (p<0.05). (B) Dual immunofluorescence for Sox17 and pHH3 was performed on lung sections from adult CCSPrtTA/tetO-Sox17 mice maintained on Dox for 3 and 5 days and positive stained cells were quantified from 20 random fields. Phospho-histone H3 was coexpressed in 28% of the Sox17-expressing respiratory epithelial cells. (0.18 MB TIF) [file pone.0005711.s001.tif]

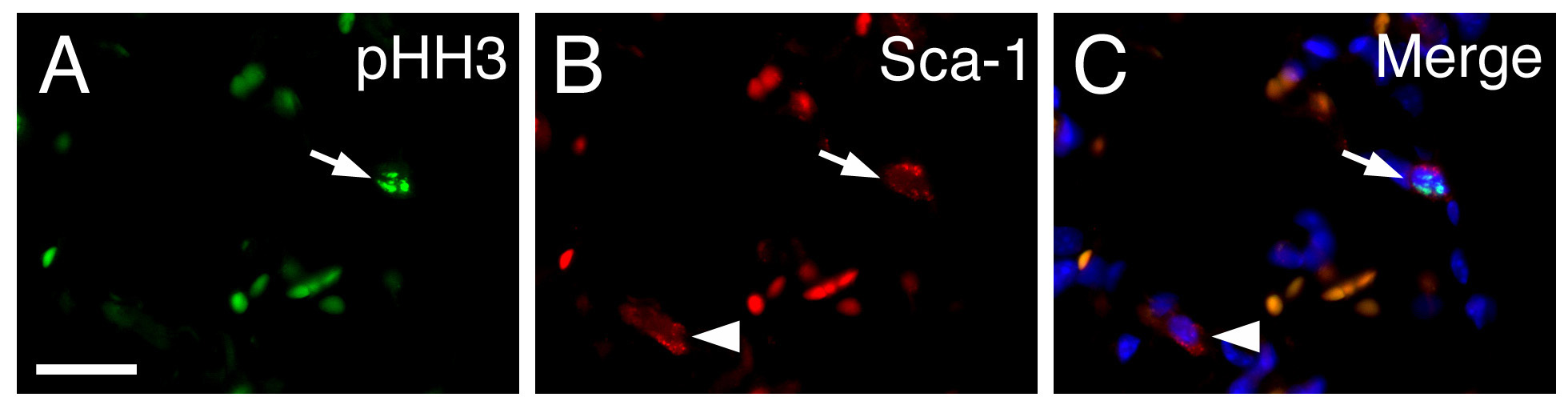

Supplement: Figure S2 — A rare subset of Sox17-induced Sca-1 positive cells coexpress phospho-histone H3. Dual-label immunofluorescence for phospho-histone H3 (pHH3; A) and Sca-1 (B) was performed on lung sections from adult CCSPrtTA/tetO-Sox17 mice maintained on Dox for 5 days. A rare subset of pHH3-positive cells (arrow) colocalized with the Sca-1-expressing cells induced by Sox17 (arrow and arrowhead; B). Nuclei are stained with DAPI. Scale bar, 20 µm. (0.36 MB TIF) [file pone.0005711.s002.tif]

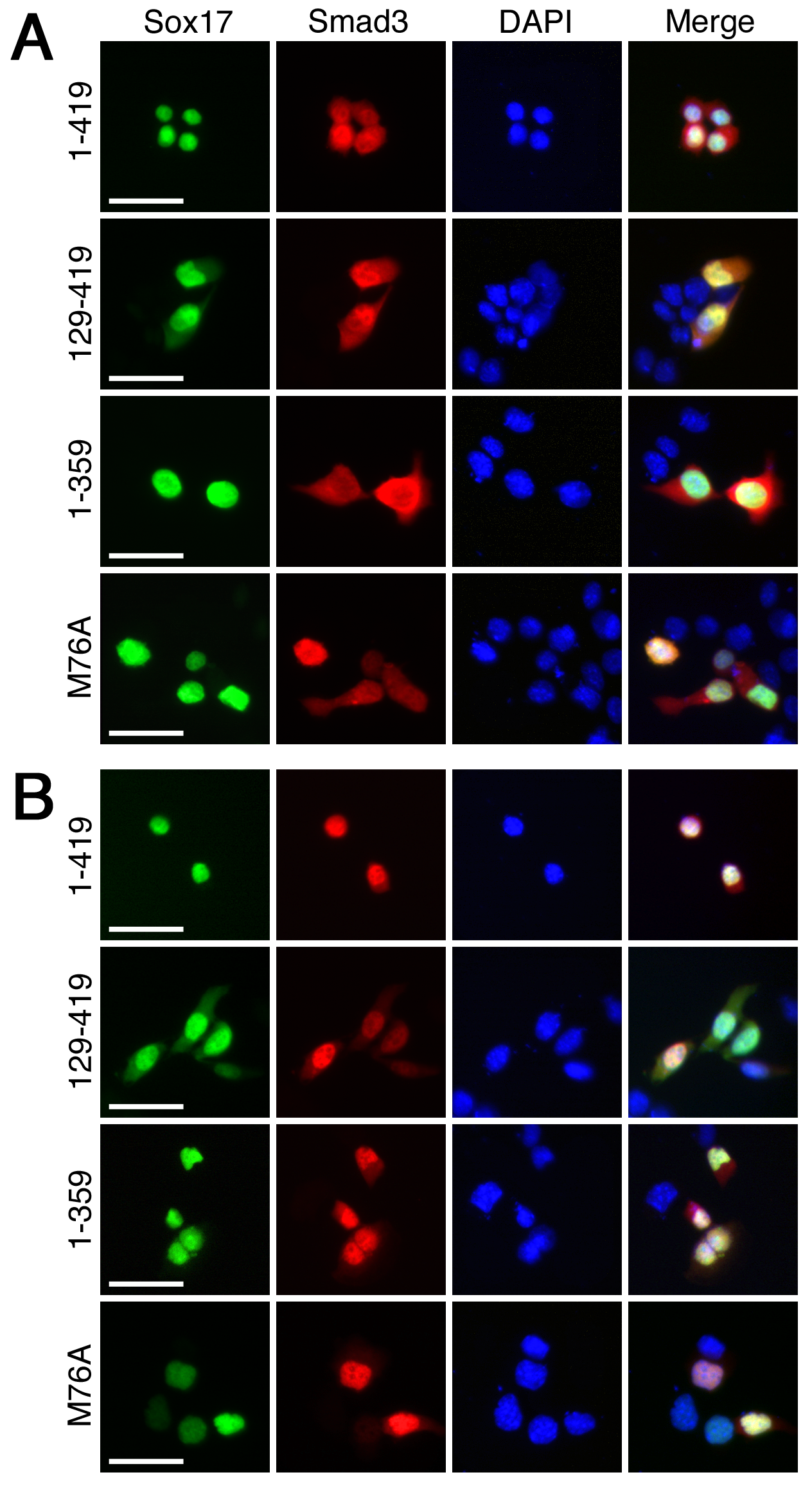

Supplement: Figure S3 — Sox17 colocalizes with Smad3 in the nucleus. MLE15 cells were transfected with FLAG-Smad3 and V5-tagged Sox17 full length or mutant contructs. After 24 h, cells were maintained in the absence (A) or presence (B) of TGF-β1 (2 ng/ml) for 2 h and dual-label immunofluorescence was performed for the V5 (green) and FLAG (red) epitopes. Expression of all of the Sox17 constructs colocalized with Smad3 in the nucleus. Nuclei are stained with DAPI (blue). Scale bar, 20 µm. (2.27 MB TIF) [file pone.0005711.s003.tif]
